# Total fluid consumption and risk of bladder cancer: a meta-analysis with updated data

**DOI:** 10.18632/oncotarget.18100

**Published:** 2017-05-23

**Authors:** Qinyu Liu, Banghua Liao, Ye Tian, Yuntian Chen, Deyi Luo, Yifei Lin, Hong Li, Kun-Jie Wang

**Affiliations:** ^1^ Urology Department, Institute of Urology (Laboratory of Reconstructive Urology), West China Hospital, Sichuan University, Chengdu, Sichuan, China; ^2^ Urology Department, Guizhou Provincial People's Hospital, Guiyang, Guizhou, China

**Keywords:** bladder cancer, risk factor, fluid consumption, epidemiology, meta-analysis

## Abstract

With meta-analysis we tented to reveal the potential relationship between daily fluid consumption and bladder cancer risk, and to find out a recommendation on daily fluid intake. Databases of the Web of Science, PubMed and EMBASE were searched then 21 case-control and 5 cohort studies were included. Stratified analyses on gender, region, time of subjects recruiting and fluid quantity were performed as well as dose-response meta-analysis. Comparing the highest exposure category with the lowest in each study, no association appeared when all data pooled together (p=0.50), but a significant OR of 1.46 (1.02-2.08, p=0.04) was found in male subgroup. For different regions, the summarized OR was 1.44 (1.10-1.89) in American case-control studies, 1.87 (1.20-2.90) in European male subgroup and 0.24 (0.10-0.60) in Asia. There was a significant relationship that each increment 1000ml daily consumption would increase the risk by 28.6% in European male (p=0.007). Similarly every additional 1000ml consumption may increase the OR by 14.9% in American people but the association wasn't that strong (p=0.057). Stratified analyses showed fluid consumption over 3000ml/day in American residents and 2000ml/day in European male resulted in OR>1 with statistical significance. In conclusion, a relationship between higher fluid intake and higher bladder cancer risk was observed in European male and American residents and a limitation to <2000ml and <3000ml per day are recommended respectively.

## INTRODUCTION

Bladder cancer is known as one of the most common malignant tumors and the risk of bladder cancer is believed to be tightly associated with life style, fluid consumption included. However, to date there still exist inconsistent conclusion on the effect of total fluid consumption on the risk. On the one hand, the urogenous contact hypothesis states that high total fluid intake can provided protective effect against bladder cancer. It believes increased fluid intake would lead to increased urine volume and urination frequency, resulting in reducing concentration of potential carcinogens in urine as well as their bladder contact time [1–3]. There are some powerful supports on this theory, including the prospective cohort study conducted by Michaud et al. which demonstrated a high fluid intake would decreased risk of bladder cancer in men [4]. On the other hand, while some source of fluid may be contaminated with carcinogens, such as chlorination byproducts and arsenic, a high-level intake may elevate the exposure of bladder epithelium to carcinogens and increase the risk of bladder cancer [5]. Apart from these, a number of recent studies found no association between total fluid intake and bladder cancer morbidity existed.

To solve this controversy, a pooled analysis and a meta-analysis were conducted in 2006 and 2014 respectively [6, 7]. The former one suggested that high fluid intake may increase the risk of bladder cancer, while the latter one indicated that greater fluid consumption would reduce the bladder cancer incidence in smokers. In addition to this contradiction, both studies give no advice on recommend fluid intake volume for potential high risk or beneficial population because of lacking detailed stratified analysis on quantity.

To explored more details on this problem, we conducted a meta-analysis which added missing or updated data to the previous meta-analyses. Also we conducted more specific subgroup analyses, aiming to again assess the relationship between total fluid intake and bladder cancer risk and try to conclude recommended fluid intake volumes for different population.

## RESULTS

### Search result and characteristics of included studies

**Figure 1:**
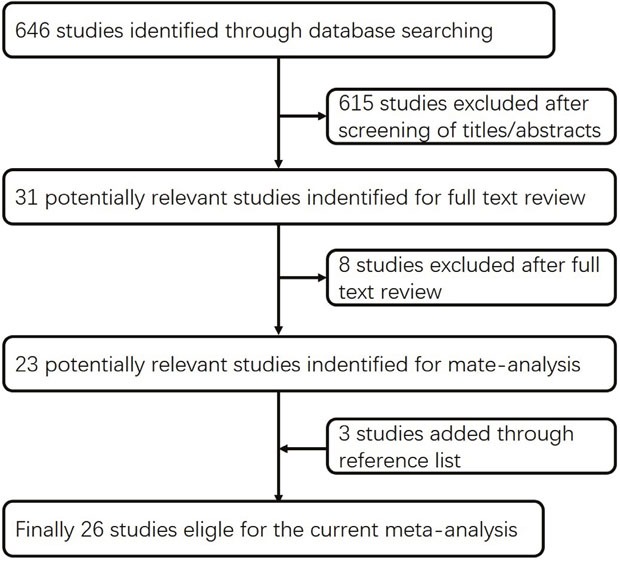
Flow diagram of the current meta-analysis

Using the predefined search strategies, 26 article were ultimately included in the current study [4, 8–32], including 21 case-control [8, 10, 12–20, 22–31] and five cohort studies [4, 9, 11, 21, 32] involving 12943 bladder cancer cases in total. Baseline characteristics of the included studies are presented in Supplementary (Supplementary Table 1 and Supplementary Table 2). The flow diagram (Figure 1) showed the detailed search steps. Among these studies, ten case-control [15, 17, 22–27, 29, 30] and two cohort studies [21, 32] reported separate outcomes of male and female. Among all studies, 10 were conducted in Europe [18–23, 27, 29, 30, 32], 11 in USA [4, 8–11, 17, 24–26, 28, 31] and 5 in Asia [12–16].

### Total fluid intake and risk of bladder cancer (highest vs lowest category given in each recruited study)

**Figure 2:**
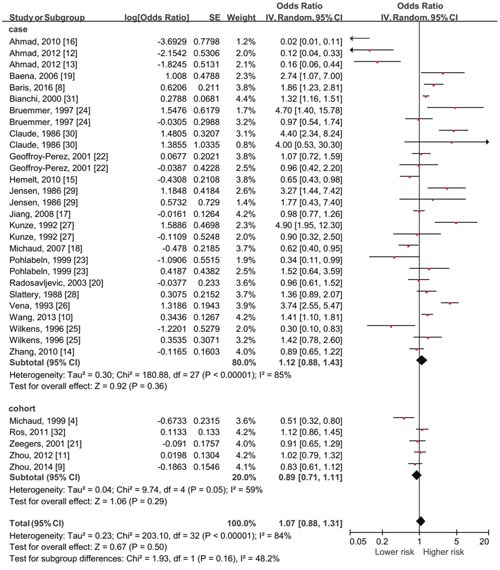
Forest plot of the association between total fluid intake and bladder cancer risk (highest vs lowest quantity in each included studies) No significant association existed with all data pooled together.

Risk estimates for high versus low level of daily fluid consumption are shown in Figure 2. The summary OR was 1.07 (using a random effects model, 95% CI: 0.88-1.31, P=0.50) of all studies, showing no statistically significant association between the highest fluid intake and the risk of bladder cancer. Significant heterogeneity showed among studies (P<0.001, I^2^=84%).

#### Total fluid intake and risk of bladder cancer by gender

**Figure 3:**
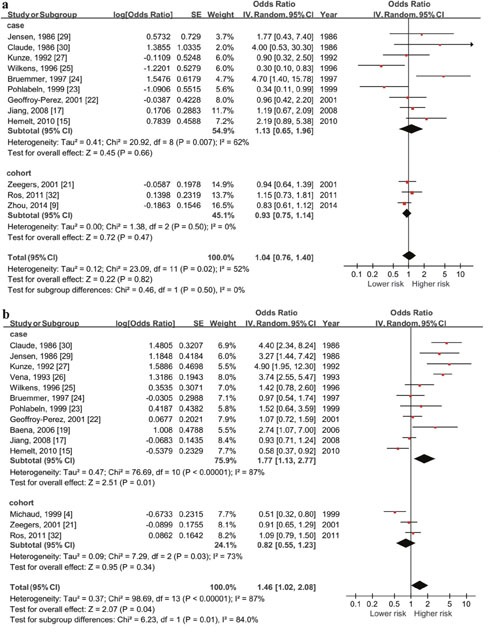
Forest plot of the association between total fluid intake and bladder cancer risk (highest vs lowest quantity) **in** female **(a)** and male **(b)**. Significant relationship between higher daily fluid intake and higher risk of bladder cancer only exist among males (OR=1.46, 95%CI=1.02-2.08, p=0.01).

The outcomes of the relationship between high-level daily fluid consumption and risk of cancer in 2 gender subgroups are shown in Figure 3. The summary OR was 1.04 (95% CI: 0.76-1.40, P=0.82) in female while was 1.46 (95% CI: 1.02-2.08, P=0.04) in male with significant association.

#### Total fluid intake and risk of bladder cancer by country (Supplementary Figure 1)

Subgroups could be divided into America, Europe and Asia according to the included studies. The summary OR of American case-control studies was 1.44 (95% CI: 1.10-1.89, P=0.009) with significant association. However when cohort study pooled in, the statistic significance disappeared (OR=1.22, 95% CI: 0.96-1.56, P=0.11). For European countries, there seemed to be a higher risk in people with high-level daily fluid consumption but the association was relatively weak (OR =1.36, 95% CI: 0.99-1.87, P=0.06). And in Asia, the summary OR was 0.78 (95% CI: 0.58-1.06, P=0.11) in China and 0.09 (95% CI: 0.03-0.24, P<0.001) in Pakistan. For all studies conducted in Asia, the pooled OR was 0.24 (95% CI: 0.10-0.60, P=0.002).

#### Total fluid intake and risk of bladder cancer by recruit time

Risk estimates for the highest versus the lowest level of total fluid consumption by recruit time subgroup are shown in Supplementary Figure 2. For those studies included in this stratified analysis, 3 of 10 studies in Europe, 4 of 10 studies in America and all studies in Asia recruited participants after 1990. The summary OR of studies recruiting participants before year 1990 was 1.28 (95% CI: 1.00-1.64, P=0.05) and 0.73 (95% CI: 0.50-1.05, P=0.09) after.

The outcomes above found that significant results were only observed among case control studies, and the pooled results were different among countries and genders. So further analyses were done for possible higher risk regions.

### Total fluid intake and bladder cancer risk in Europe

#### Total fluid intake and risk of bladder cancer in Europe by gender (Figure 4a & 4b)

As the outcomes showed, the summary OR in European female was 0.98 (95% CI: 0.73-1.32, P=0.91) and 1.87 (95% CI: 1.20-2.90, P=0.006) in male. Significant association only existed in male.

#### Total fluid intake and risk of bladder cancer in European male by daily fluid intake quantity level

**Figure 4:**
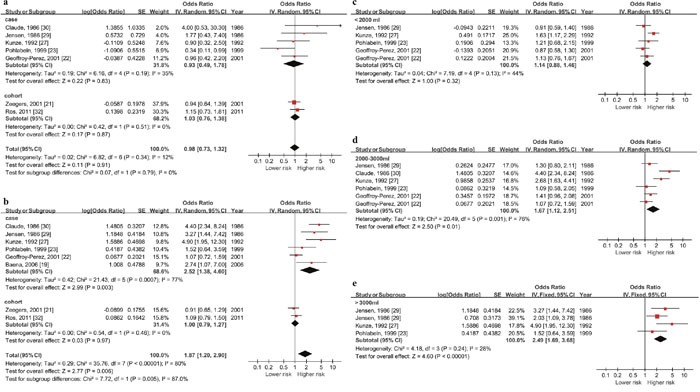
Forest plot of the association between total fluid intake and bladder cancer risk in Europe **(a)** & **(b)** show the highest vs lowest quantity outcomes in European female and male, significant relationship between higher daily fluid intake and higher risk of bladder cancer only exist among males (OR=1.87, 95%CI=1.20-2.90, p<0.001). The others figures show outcomes in European male by daily fluid intake quantity level (**(c)** >around 3000ml, **(d)** around 2000-3000ml, **(e)** <around 2000ml). Significant association show up when the level reach over 2000ml.

Since positive pooled outcome showed up in case-control studies among European males, we did a stratified analysis by daily fluid intake quantity level and dose-response meta-analysis for this population (5 studies with 1973 cases and 2386 controls). According to the included studies, daily quantity (except the reference quantity) could be divide in to the following levels: the highest quantity (>3000ml), the 2nd highest quantity (2000-3000ml) and the 3rd highest quantity (<2000ml). The summary OR of these three subgroups were 2.49 (95% CI: 1.69-3.68, P<0.001), 1.67 (95% CI: 1.12-2.51, P=0.01) and 1.14 (95% CI: 0.88-1.46, P=0.32) respectively. Significant association existed when daily quantity reached over 2000ml (Figure 4c, 4d & 4e).

The dose-response meta-analysis included 4 researches (1633 cases and 2046 controls) and showed that association between cancer risk and fluid consumption matched a liner relation (p=0.007). Each increment 1000ml daily consumption would increase the risk by 28.6% (OR=1.286, 95% CI=1.071-1.544). No evidences of non-linear relationship existed. (p=0.438) (Figure 6a).

**Figure 5:**
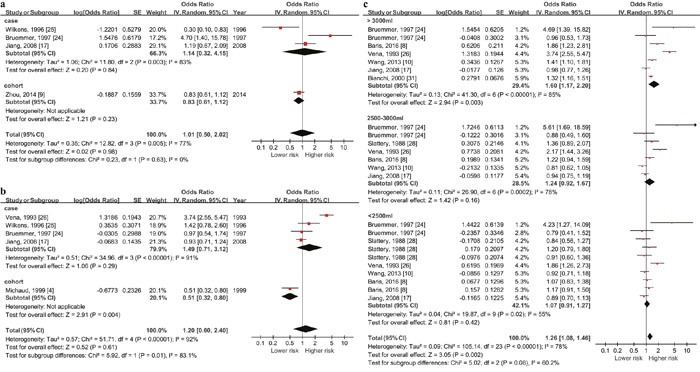
Forest plot of the association between total fluid intake and bladder cancer risk in America **(a)** & **(b)** show the highest vs lowest quantity outcomes in European female and male and **(c)** shows outcomes in American residents by daily fluid intake quantity level (>3000ml, 2500-3000ml and <2500ml). Significant association show up when the level reach over 3000ml with the data from both genders.

**Figure 6:**
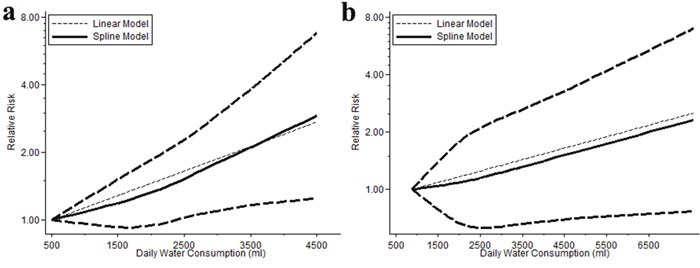
Dose-response meta-analysis outcomes of data from European male and American people of both gender There is a strong relationship in European males that each increment 1000ml daily consumption would increase the risk by 28.6% (p=0.007) **(a)**, and a relatively weaker association in American people that every additional 1000ml daily consumption may increase the OR by 14.9% (p=0.057) **(b)**.

### Total fluid intake and bladder cancer risk in America

#### Total fluid intake and risk of bladder cancer in America by gender (Figure 5a & 5b)

The summary OR was 1.14 (95% CI: 0.32-4.15, P=0.84) in American female and 0.83 (95% CI: 0.61-1.12, P=0.23) in America male. No statistic significance existed in both gender.

#### Total fluid intake and risk of bladder cancer in America by daily fluid intake quantity level

Since American case-control studies showed higher risk in high fluid intake population, we did a stratified analysis by daily fluid intake quantity level using the data of both genders from case control studies (6 studies with 4636 cases and 6274 controls). Since upper and lower boundaries in each category varied greatly in included studies, daily quantity was divided into the following levels according to the midpoint value of each category: the highest quantity (>3000ml), the 2nd highest quantity (2500-3000ml) and the 3rd highest quantity (<2500ml). The summary OR of these three subgroups were 1.60 (95% CI: 1.17-2.20, P<0.001), 1.24 (95% CI: 0.92-1.67, P=0.16) and 1.07 (95% CI: 0.91-1.27, P=0.42) respectively (Figure 5c). Significant association existed when daily quantity >3000ml.

Dose-response meta-analysis of these 6 studies showed no evidence of nonlinear relationship (p=0.772). Linear analysis suggested that every additional 1000ml consumption would increase the OR by 14.9% (OR=1.149, 95% CI=0.996-1.326) but without statistic significance (p=0.057) (Figure 6b).

### Publication bias

There was no asymmetry in funnel plot for the association between total fluid intake and risk of bladder cancer (Supplementary Figure 3). P values for Begg's adjusted rank correlation test was 0.422, and the Egger's regression asymmetry test was 0.243, suggesting that no significant publication bias existed in this meta-analysis.

## DISCUSSION

To our knowledge, the current study represents the most comprehensive and up-to-date review on this topic. In this review and meta-analysis of 26 studies with detailed stratified analyses, we showed that there was no unified association between total fluid consumption and the risk of bladder cancer for all people. Nevertheless, high fluid intake would obviously increase the risk in European male and this relationship might also lie in American residents but was relatively weak. On the contrary however, a protective effect from high fluid intake against bladder cancer was noticed in Asia population, especially in Pakistan. The results indicated that the association between total fluid intake and risk of bladder cancer was largely affected by differences in gender, geography region and even the time when the participants were included. Basing on the findings of the current study and those of other previous related studies, we inferred that it was the source and quality of water as well as diets that much related to bladder cancer risk while volume of fluids consumption played a less important role. Our study also provided the evidence that male would be more easily affected by these environmental and living habit factor than female. To prevent higher risk, according to the evidences found in the current study, it is suggested to limit total daily fluid consumption to 2000ml for European males and 3000ml for America residents. There is a significant relationship that every increment 1000ml daily consumption would increase the risk by 28.6% in European male. Similarly every additional 1000ml consumption may increase the OR by 14.9% in American people but the association is not that strong.

What is noteworthy, however, is that the majority of data from Europe and America was collected from cases included before 1990, which may be unable to represent the present conditions. Subgroup analysis by time in the current study indicated that when pooled together, cases recruited before 1990 showed positive outcomes while more recent studies provided negative ones. This difference may mainly generate from the evolution in disinfection techniques and regulations. Taking America for example, since the first regulation on disinfection by-products was passed in 1979, chemical substances including trihalomethanes and free residual chlorine have been successively monitored [33]. Usage of arsenical also keeps declining over the years [8]. All these substances mentioned above are believed to be potential carcinogens for bladder cancer [34–37]. With the improvement of water quality, the increase risk of bladder cancer brought by water pollution is presently better under control. As a result, for the included studies, the conclusions on association between total water intake and risk of bladder cancer altered from positive to negative as time went on.

Another point to be noted is that significant differences were only observed in case-control studies and the outcome varied among regions. Total water intake had a greater impact on residents in European and America than in Asia. Apart from the potential effects of ethnic difference, diet habits, components of daily fluid intake more specifically, might also played important roles. The Chinses are used to drink boiled water, which may further eliminate organic residues in water and reduce the harm taken by them. Furthermore, as one of the most popular beverages in Asia, tea is proved to have protective effect on several cancers, bladder cancer included [38]. Polyphenols in tea was found able to provide protection against bladder cancer by antioxidant activity [39]. These may partly explain why high fluid intake had little impact or even protective effect on risk of bladder cancer in Asian population. On the other hand, tap water is one of the major source of water for people in America and European countries and it has been proved that intake of tap water may induce increasing risk of bladder cancer [6]. In addition, coffee, as one of the major beverages consumed in Europe and America, may also contribute an increased risk of bladder cancer [26, 40, 41]. It was interesting to notice that Wu et al. [40] demonstrated in their study that male coffee drinkers were more likely to develop bladder cancer and Cantor et al. [41] found the trend in bladder cancer risk from tap water was significant for men only. These outcomes matched our finding that the males were more susceptible to environmental and diet risk factors.

Recently, Buendia et al. [42] observed a slight decrease in urinary adducts formation and significantly decreased urinary mutagenicity with increasing water intake in male smoker, which firstly proved urogenous contact hypothesis. So for the purpose of preventing bladder cancer in European and American population, apart from limiting intake volume as mentioned above without changing drinking style, try to drink more non-tap water as well as tea may also help.

Altieri et al. [43] and Bai et al. [7] also conducted meta-analysis on the same topic previously. Altieri et al. explored fluid intake and risk of other cancers as well which made the topic more extensive. But since there were at least 11 more related studies published after his work, his conclusion may unable to keep up to date. Bai et al. conducted their study in 2014 and their did much work on subgroup meta-analysis for different types of beverages and smoke status. However, they missed some important published data and just concluded a sweeping statement but didn't give any specific advice. Our study identified significantly 5 more studies, including those missed by Bai and newly published ones. Because the updated studies provided no detailed data on specific beverages or smoke status, we didn't repeated Bai's work. We focused on subgroup analysis by different exposure level in different gender of population in different region and finally summarized a recommendation list of total fluid intake for preventing increasing risk of bladder cancer.

The limitations of this meta-analysis should be acknowledged. First, only published data were included, making publication bias particularly inevitable even though no significant evidence was observed by statistic analysis. Second, we found that the majority of the data in included studies were collected from cohort or diagnosed cases recruited over 25 years ago, which may unable to reflect the exact conditions of the present. More well-designed studies are warranted to draw more proper conclusions.

In conclusion, the current study indicates that high fluid intake seems to be a risk factor for bladder cancer in European male and American residents but a protective effect in Asian. Geography region seem to be stronger influence factor on fluid intake and risk of bladder cancer. The reason may lie in differences in water quality and drinking habits. Male are proved to be more susceptible to these potential risk factors. For protective purpose, a recommended total fluid consumption is listed as followed: limitation to 3000ml per day for people living in America and <2000ml per day for European males are recommended. Drinking more boiled water and tea may also help. The actual recommended volume may be higher than mentioned above because a large part of the included data were collected in the past time when water quality wasn't managed as well as the present. So more new case-control and cohort studies are needed to draw more proper conclusion.

## MATERIALS AND METHODS

The study was designed under the guidance of the Meta-Analysis of Observational Studies in Epidemiology guidelines for study reporting [44].

### Literature search

We searched the Web of Science, PubMed and EMBASE to identify articles published up to June 2016. Search terms included “fluid or water” and “intake or consumption” and “bladder cancer or urothelial cancer or transitional or bladder neoplasm or bladder carcinoma”. No language limitations were imposed.

### Inclusion criteria

Inclusion criteria were as followed: (1) case-control or cohort study exploring the relationship between total fluid consumption and bladder cancer risk, (2) provided exact data in both case and control groups (participants for cohort studies) and (3) adjusted effect estimates with their 95% CIs were directly given or could be calculated from the present data.

Studies with overlapping or insufficient data were excluded.

### Selection and data extraction

Two authors (LQ and TY) performed the selection work. Screening was done by titles and abstracts reading. Full text were reviewed when the abstract was insufficient to determine if the study met the inclusion or exclusion criteria. The references of all studies included were manually searched to identify additional studies. Final agreement on inclusion was made by consensus with all authors. All data, including evaluation of study characteristics, risk of bias, and outcome measures, were extracted by two authors.

### Methodological quality assessment

The Newcastle-Ottawa Scale (NOS) [45] was used to assess the quality of included studies. Two authors scored these studies independently and final result was made by discussion and consensus with a third author if disagreement came up. Studies that scored >7 were considered as having low risk of bias while scores of <5 indicated high risk of bias.

### Statistical analysis

The ORs were extracted and transformed to their natural logs for each include study. The log ORs were weighted by the inverse of their variances to obtain a pooled OR with 95% CI. The Q-test was adopted for statistical heterogeneity measured [46] and the I^2^ score calculating [47]. Heterogeneity was considered present when P<0.10. I^2^ >50% was considered as presence of heterogeneity. In cases lacking of heterogeneity, the Mantel-Haenszel fixed-effect model was used to provide summary estimations, otherwise, the DerSimonian and Laird random-effect model was adopted for the meta-analysis [46, 48]. A dose-response meta-analysis was done according to given data.

Publication bias was assessed through funnel plots as well as tests of Begg rank correlation and Egger regression asymmetry. P<0.05 was considered to be representative of a significant statistical publication bias. Subgroup analyses were conducted as followed to explore the potential heterogeneity among studies: gender (male and female), study design (cohort and case-control studies), geography region (Europe, America and Asia), cases recruited year (before 1990 and after 1990), Fluid intake quantity (selected based on the characteristics of included studies).

Statistical significance was set at P<0.05. STATA version 12.0 (StataCorp, College Station, Texas, United States) and Review Manager 5.2 (The Nordic Cochrane Centre, The Cochrane Collaboration, 2012) was used for the statistical analyses.

## SUPPLEMENTARY MATERIALS FIGURES AND TABLES




