# Supplementary material for: Total fluid consumption and risk of bladder cancer: a meta-analysis with updated data
Source: Oncotarget. 2017 May 23;8(33):55467–77. doi: 10.18632/oncotarget.18100 (PMC5589673; doi:10.18632/oncotarget.18100)
Supplement: Supplementary file 1 [file oncotarget-08-55467-s001.pdf]

Total fluid consumption and risk of bladder cancer: a meta-analysis with updated data

SUPPLEMENTARY MATERIALS

Supplementary Table 1: Study characteristics of published cohort studies of total fluid consumption and bladder cancer risk

See Supplementary File 1

Supplementary Table 2: Study characteristics of published case–control studies of total fluid intake and bladder cancer risk

See Supplementary File 1

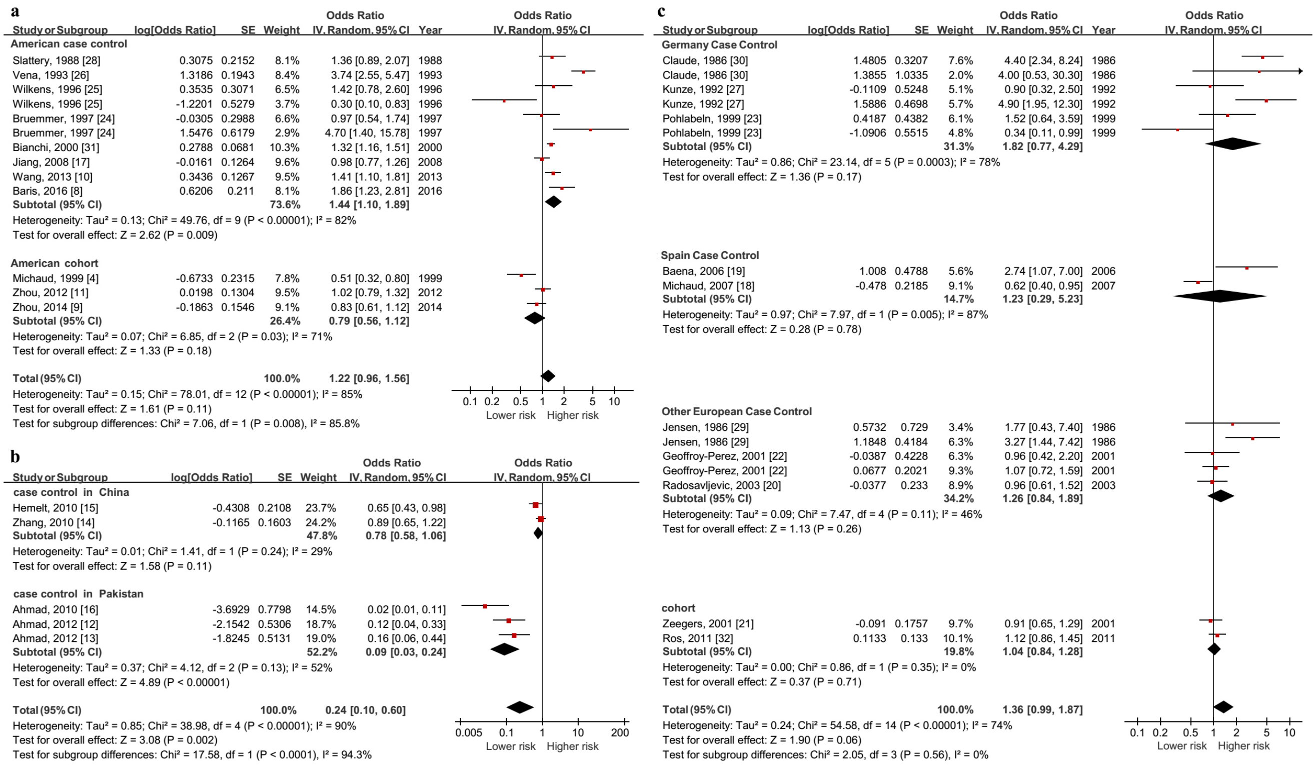

Supplementary Figure 1: Forest plot of the association between total fluid intake and bladder cancer risk (highest vs lowest quantity) in America **(a),** Asia **(b)** and Europe **(c).**

Study or Subgroup	log[Odds Ratio]	SE	Weight	Odds Ratio IV, Random, 95% CI	Year
**Recruited before (in) 1990**					
Claude, 1986 [30]	1.4805	0.3207	3.0%	4.40 [2.34, 8.24]	1986
Claude, 1986 [30]	1.3855	1.0335	0.8%	4.00 [0.53, 30.30]	1986
Jensen, 1986 [29]	0.5732	0.729	1.4%	1.77 [0.43, 7.40]	1986
Jensen, 1986 [29]	1.1848	0.4184	2.5%	3.27 [1.44, 7.42]	1986
Slattery, 1988 [28]	0.3075	0.2152	3.6%	1.36 [0.89, 2.07]	1988
Kunze, 1992 [27]	-0.1109	0.5248	2.0%	0.90 [0.32, 2.50]	1992
Kunze, 1992 [27]	1.5886	0.4698	2.3%	4.90 [1.95, 12.30]	1992
Vena, 1993 [26]	1.3186	0.1943	3.7%	3.74 [2.55, 5.47]	1993
Wilkens, 1996 [25]	-1.2201	0.5279	2.0%	0.30 [0.10, 0.83]	1996
Wilkens, 1996 [25]	0.3535	0.3071	3.1%	1.42 [0.78, 2.60]	1996
Bruemmer, 1997 [24]	-0.0305	0.2988	3.2%	0.97 [0.54, 1.74]	1997
Bruemmer, 1997 [24]	1.5476	0.6179	1.7%	4.70 [1.40, 15.78]	1997
Michaud, 1999 [4]	-0.6733	0.2315	3.5%	0.51 [0.32, 0.80]	1999
Pohlabeln, 1999 [23]	0.4187	0.4382	2.4%	1.52 [0.64, 3.59]	1999
Pohlabeln, 1999 [23]	-1.0906	0.5515	1.9%	0.34 [0.11, 0.99]	1999
Bianchi, 2000 [31]	0.2788	0.0681	4.3%	1.32 [1.16, 1.51]	2000
Geoffroy-Perez, 2001 [22]	0.0677	0.2021	3.7%	1.07 [0.72, 1.59]	2001
Zeegers, 2001 [21]	-0.091	0.1757	3.8%	0.91 [0.65, 1.29]	2001
Geoffroy-Perez, 2001 [22]	-0.0387	0.4228	2.5%	0.96 [0.42, 2.20]	2001
Ros, 2011 [32]	0.1133	0.133	4.0%	1.12 [0.86, 1.45]	2011
Zhou, 2012 [11]	-0.2718	0.1228	4.1%	0.76 [0.60, 0.97]	2012
Zhou, 2014 [9]	-0.2231	0.1641	3.9%	0.80 [0.58, 1.10]	2014
**Subtotal (95% CI)**			**63.5%**	**1.28 [1.00, 1.64]**	
Heterogeneity: Tau² = 0.22; Chi² = 124.66, df = 21 (P < 0.00001); I² = 83%					
Test for overall effect: Z = 2.00 (P = 0.05)					
**Recruited after 1990**					
Radosavljevic, 2003 [20]	-0.0377	0.233	3.5%	0.96 [0.61, 1.52]	2003
Baena, 2006 [19]	1.008	0.4788	2.2%	2.74 [1.07, 7.00]	2006
Michaud, 2007 [18]	-0.478	0.2185	3.6%	0.62 [0.40, 0.95]	2007
Zhang, 2010 [14]	-0.1165	0.1603	3.9%	0.89 [0.65, 1.22]	2010
Hemelt, 2010 [15]	-0.4308	0.2108	3.7%	0.65 [0.43, 0.98]	2010
Ahmad, 2010 [16]	-3.6929	0.7798	1.2%	0.02 [0.01, 0.11]	2010
Ahmad, 2012 [12]	-2.1542	0.5306	2.0%	0.12 [0.04, 0.33]	2012
Zhou, 2012 [11]	-0.0305	0.1245	4.1%	0.97 [0.76, 1.24]	2012
Ahmad, 2012 [13]	-1.8245	0.5131	2.1%	0.16 [0.06, 0.44]	2012
Wang, 2013 [10]	0.3436	0.1267	4.1%	1.41 [1.10, 1.81]	2013
Zhou, 2014 [9]	0.077	0.4344	2.4%	1.08 [0.46, 2.53]	2014
Baris, 2016 [8]	0.6206	0.211	3.7%	1.86 [1.23, 2.81]	2016
**Subtotal (95% CI)**			**36.5%**	**0.73 [0.50, 1.05]**	
Heterogeneity: Tau² = 0.31; Chi² = 81.40, df = 11 (P < 0.00001); I² = 86%					
Test for overall effect: Z = 1.70 (P = 0.09)					

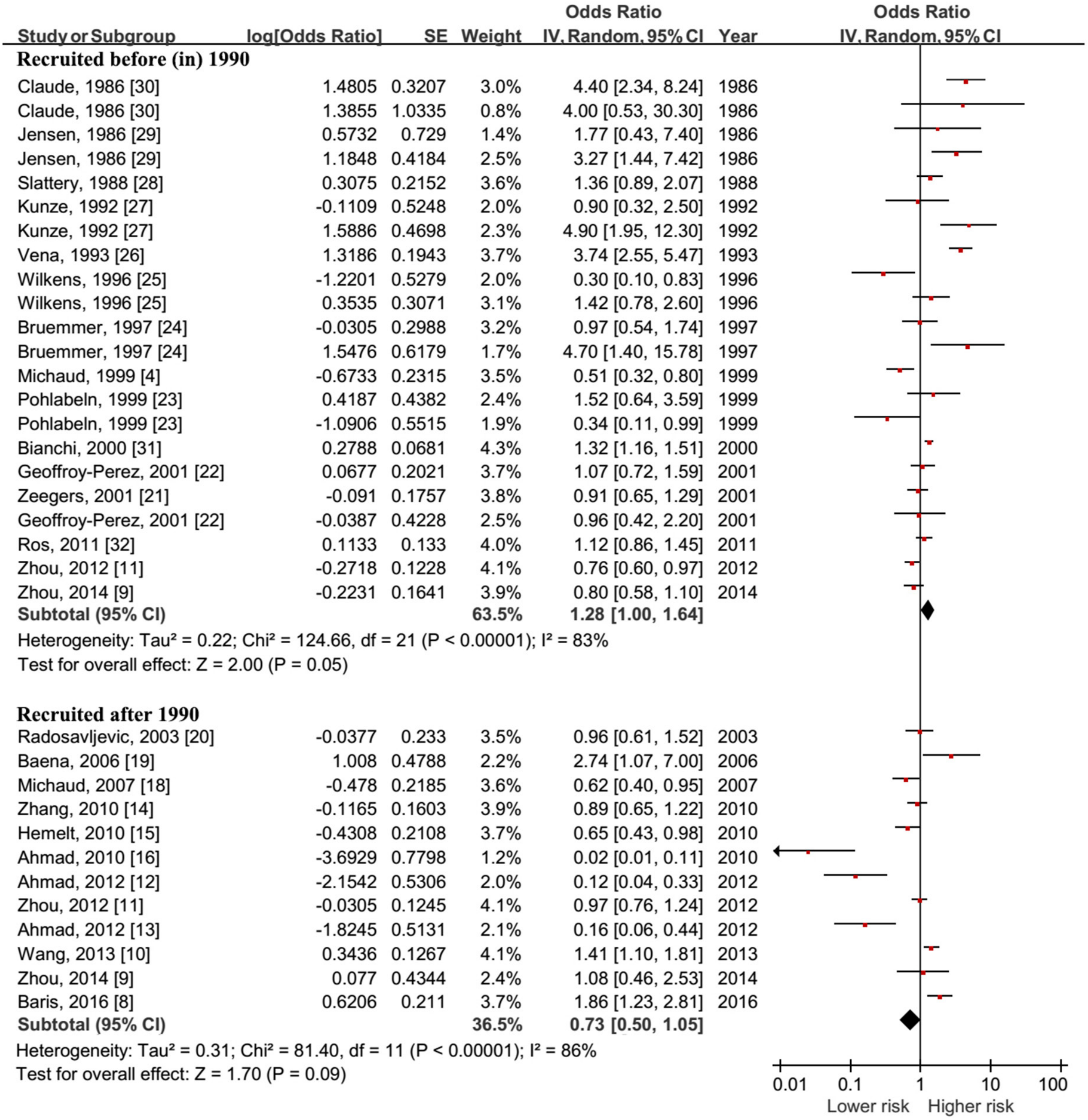

Supplementary Figure 2: Forest plot of the association between total fluid intake and bladder cancer risk (highest vs lowest quantity) in studies recruiting participants before (in) 1990 and after 1990.

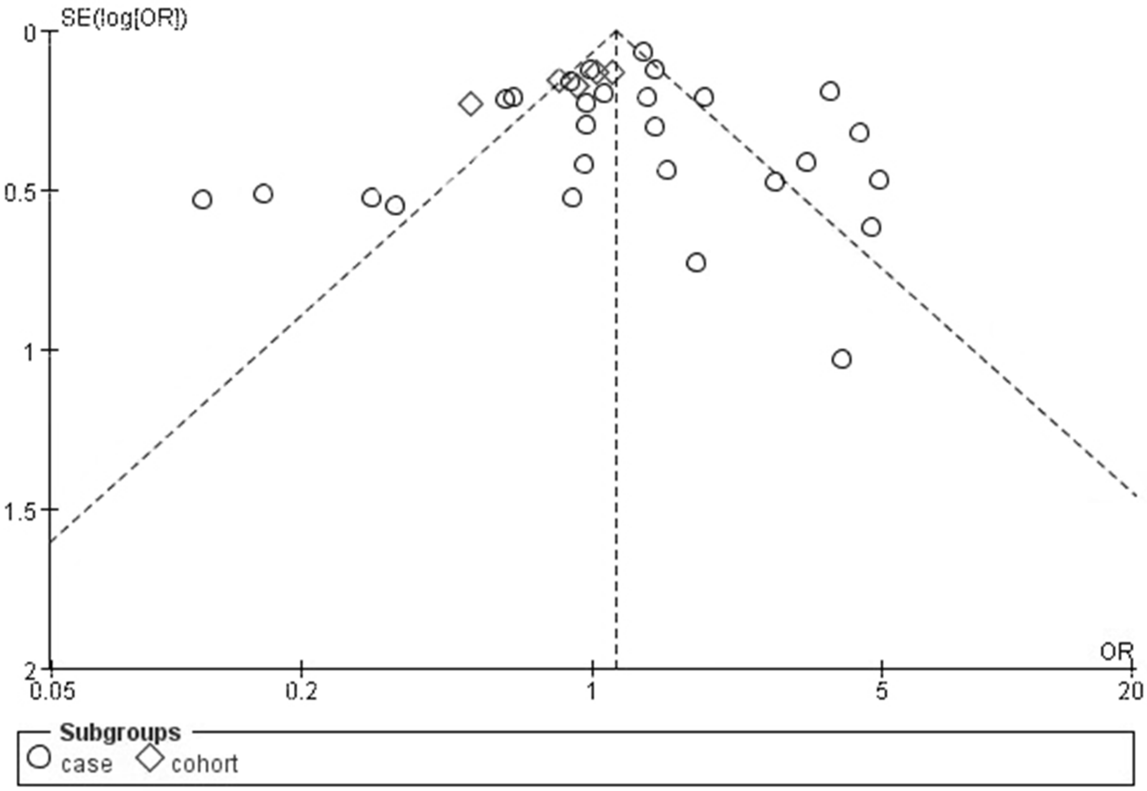

Supplementary Figure 3: Funnel plot of studies included evaluating the association between total fluid consumption and bladder cancer risk.
