# Supplementary material for: Total fluid consumption and risk of bladder cancer: a meta-analysis with updated data
Source: Oncotarget. 2017 May 23;8(33):55467–77. doi: 10.18632/oncotarget.18100 (PMC5589673; doi:10.18632/oncotarget.18100)
Supplement: Supplementary file 2 [file oncotarget-08-55467-s002.doc]

| **Supplementary Table 1: Study characteristics of published cohort studies of total fluid consumption and bladder cancer risk** | | | | | | | | | | |
| --- | --- | --- | --- | --- | --- | --- | --- | --- | --- | --- |
| Source | | Country | Sex | Follow-up duration  (years) | | Total No./ No. of Cases | Sub-  group | Total fluid consumption (ml/day) | Adjusted OR (95% CI) | Study quality |
| Michaud, 1999 | | USA | M | | 10 | 47,909/252 | M | <1290 | 1.0 | 8 |
|  | |  |  | |  |  |  | 1290–1674 | 0.84 (0.58–1.21) |  |
|  | |  |  | |  |  |  | 1675–2050 | 0.89 (0.62–1.29) |  |
|  | |  |  | |  |  |  | 2051–2531 | 0.70 (0.47–1.04) |  |
|  | |  |  | |  |  |  | >2531 | 0.51 (0.32–0.80) |  |
| Zeegers, 2001 | | Netherlands | F/M | | 6.3 | 120,852/569 | F | 340-1686 | 1.00 | 7 |
|  | |  |  | |  |  |  | 1687-1916 | 0.80(0.57-1.11) |  |
|  | |  |  | |  |  |  | 1917-2130 | 0.86(0.61-1.21) |  |
|  | |  |  | |  |  |  | 2131-2435 | 0.91(0.63-1.30) |  |
|  | |  |  | |  |  |  | 2436-5352 | 0.94(0.64-1.39) |  |
|  | |  |  | |  |  | M | 12-984 | 1.00 |  |
|  | |  |  | |  |  |  | 985-1181 | 0.83(0.55-1.25) |  |
|  | |  |  | |  |  |  | 1181-1372 | 0.74(0.48-1.13) |  |
|  | |  |  | |  |  |  | 1373-1614 | 1.04(0.69-1.56) |  |
|  | |  |  | |  |  |  | 1615-4647 | 0.91(0.65-1.29) |  |
|  | |  |  | |  |  | Both | 12-984 | 1.00 |  |
|  | |  |  | |  |  |  | 985-1181 | 0.83 (0.55-1.25) |  |
|  | |  |  | |  |  |  | 1181-1372 | 0.74 (0.48-1.13) |  |
|  | |  |  | |  |  |  | 1373-1614 | 1.04 (0.69-1.56) |  |
|  | |  |  | |  |  |  | 1615-4647 | 0.91 (0.65-1.29) |  |
| Ros, 2011 | | European | F/M | | 9.3 | 233,236/513 | F | <1,438 | 1.0 | 8 |
|  | |  |  | |  |  |  | 1,438–2,046 | 0.92 (0.60–1.42) |  |
|  | |  |  | |  |  |  | >2,046 | 1.15 (0.73–1.81) |  |
|  | |  |  | |  |  | M | <1,735 | 1.0 |  |
|  | |  |  | |  |  |  | 1,735–2,425 | 1.13 (0.86–1.48) |  |
|  | |  |  | |  |  |  | >2,425 | 1.09 (0.79–1.50) |  |
|  | |  |  | |  |  | Both | Q1 | 1 |  |
|  | |  |  | |  |  |  | Q2 | 1.07 (0.85–1.35) |  |
|  | |  |  | |  |  |  | Q3 | 1.12 (0.86–1.45) |  |
| Zhou, 2012 | | USA | F/M | | 22 | 924,221/823 | 1986 diet | <1,290 | 1.0 | 7 |
|  | |  |  | |  |  |  | 1,290–1,674 | 0.96(0.78,1.19) |  |
|  | |  |  | |  |  |  | 1,675–2,050 | 0.93(0.74,1.15) |  |
|  | |  |  | |  |  |  | 2,051–2,531 | 0.80(0.64,1.01) |  |
|  | |  |  | |  |  |  | >2,531 | 0.76(0.60,0.97) |  |
|  | |  |  | |  |  | most rencent | <1,290 | 1.0 |  |
|  | |  |  | |  |  |  | 1,290–1,674 | 1.17(0.94,1.45) |  |
|  | |  |  | |  |  |  | 1,675–2,050 | 1.08(0.86,1.35) |  |
|  | |  |  | |  |  |  | 2,051–2,531 | 1.01(0.80,1.27) |  |
|  | |  |  | |  |  |  | >2,531 | 0.97(0.76,1.24) |  |
|  | |  |  | |  |  | cumulative | <1,290 | 1.0 |  |
|  | |  |  | |  |  |  | 1,290–1,674 | 1.13(0.90,1.41) |  |
|  | |  |  | |  |  |  | 1,675–2,050 | 1.24(0.99,1.55) |  |
|  | |  |  | |  |  |  | 2,051–2,531 | 0.98(0.77,1.25) |  |
|  | |  |  | |  |  |  | >2,531 | 1.02(0.79,1.32) |  |
| Zhou. 2014 | | USA | F | | 20 | 160,041/427 | F | Q1 | 1.0 | 7 |
|  | |  |  | |  |  | F | Q2 | 1.26 (0.83, 1.92) |  |
|  | |  |  | |  |  | F | Q3 | 0.95 (0.71, 1.26) |  |
|  | |  |  | |  |  | F | Q4 | 0.83 (0.61, 1.12) |  |
|  | Both: both gender; Q: quantity level. | | | | | | | | | |

| **Supplementary Table 2: Study characteristics of published case–control studies of total fluid intake and bladder cancer risk** | | | | | | | |
| --- | --- | --- | --- | --- | --- | --- | --- |
| Source | Country | Sex | Number of cases/ controls | Subgroup | Total fluid consumption | Adjusted OR (95% CI) | Study quality |
| Claude, 1986 | Germany | M/F | 431/431 | M | >2.0 vs.<2. 0 L/day | 4.4(2.3-8.2) | 6 |
|  |  |  |  | F | >2.0 vs.<2. 0 L/day | 4.0(0.5-30.3) |  |
| Jensenet al. 1986 | Denmark | M/F | 371/771 | M | 0-0.99 L/d | 1.0 | 7 |
|  |  |  |  |  | 1-1.99 L/day | 0.9(0.6,1.4) |  |
|  |  |  |  |  | 2-2.99 L/day | 1.3(0.8,2.1) |  |
|  |  |  |  |  | 3-3.99 L/day | 2.0(1.1,3.8) |  |
|  |  |  |  |  | 4+ L/day | 3.3(1.4,7.4) |  |
|  |  |  |  | F | 0-0.99 L/day | 1.0 |  |
|  |  |  |  |  | 1-1.99 L/day | 1.1(0.6.1.9) |  |
|  |  |  |  |  | 2-2.99 L/day | 1.3(0.5,3.2) |  |
|  |  |  |  |  | 3-3.99 L/day | 1.8(0.4,7.4) |  |
| Slattery, 1988 | USA | M/F | 419/889 | / | ≤289 ozs/week | 1.0 | 6 |
|  |  |  |  |  | 290-387 ozs/week | 0.91(0.60-1.36) |  |
|  |  |  |  |  | 388-488 ozs/week | 0.84(0.56-1.27) |  |
|  |  |  |  |  | 489-653 ozs/week | 1.20(0.79-1.80) |  |
|  |  |  |  |  | >653 ozs/week | 1.36(0.89-2.07) |  |
| Kunze, 1992 | Germany | F/M | F:75/71 | F | 1.1-2.0 L/day | 1.2(0.6-2.1) | 6 |
|  |  |  |  |  | 2.1-3.0 L/day | 0.9(0.3-2.5) |  |
|  |  |  | M:416/360 | M | 1.1-2.0 L/day | 1.6(1.2-2.3) |  |
|  |  |  |  |  | 2.1-3.0 L/day | 2.7(1.6-4.4) |  |
|  |  |  |  |  | 3.1+ L/day | 4.9(2.0-12.3) |  |
| Vena, 1993 | USA | M | 351/855 | M | 2-7 cups/day | 1.00 | 8 |
|  |  |  |  |  | 8-10 cups/day | 1.86(1.26-2.73) |  |
|  |  |  |  |  | 11-13 cups/day | 2.17(1.44-3.26) |  |
|  |  |  |  |  | 14-49 cups/day | 3.74(2.55-5.47) |  |
| Wilkenset al. 1996 | USA | F/M | F: 66/132 | F | Q1 | 1.0 | 7 |
|  |  |  |  |  | Q2 | 0.6 (0.3-1.5) |  |
|  |  |  |  |  | Q3 | 0.4 (0.2-1.1) |  |
|  |  |  |  |  | Q4 | 0.3 (0.1-0.8) |  |
|  |  |  | M: 195/390 | M | Q1 | 1.0 |  |
|  |  |  |  |  | Q2 | 1.2 (0.7-2.1) |  |
|  |  |  |  |  | Q3 | 1.2 (0.7-2.0) |  |
|  |  |  |  |  | Q4 | 1.4 (0.8-2.6) |  |
| Bruemmeret al. 1997 | USA | F/M |  | F | <7 cups/day (8ounces or 240ml/cup) | 1.0 | 6 |
|  |  |  |  |  | >7-9 cups/day | 4.2（1.3-14.1） |  |
|  |  |  |  |  | >9-12 cups/day | 5.6（1.7-18.6） |  |
|  |  |  |  |  | >12 cups/day | 4.7（1.4-15.8） |  |
|  |  |  |  | M | <7 cups/day | 1.0 |  |
|  |  |  |  |  | >7-9 cups/day | 0.8（0.4-1.5） |  |
|  |  |  |  |  | >9-12 cups/day | 0.9（0.5-1.6） |  |
|  |  |  |  |  | >12 cups/day | 1.0（0.5-1.7） |  |
| Pohlabeln, 1999 | Germany | F/M | F:61/61 | F | 0-1 L/day | 1.0 | 6 |
|  |  |  |  |  | 1-2 L/day | 0.59(0.25-1.39) |  |
|  |  |  |  |  | 2+ L/day | 0.34(0.11-0.99) |  |
|  |  |  | M:239/239 | M | 0-1 L/day | 1.0 |  |
|  |  |  |  |  | 1-2 L/day | 1.21(0.68-2.15) |  |
|  |  |  |  |  | 2-3 L/day | 1.09(0.58-2.05) |  |
|  |  |  |  |  | 3+ L/day | 1.52(0.64-3.59) |  |
| Bianchi et al. 2000 | USA | F/M | 1,452/2,434 |  | ≥2.6 vs. <2.6 L/day | 1.32 (1.16-1.51) | 7 |
| Geoffroy-Perez. | France | F/M | F:106/106 | F | 0–7300 ml/week | 1.0 | 6 |
| 2001 |  |  |  |  | 7301–9900 ml/week | 0.70(0.30–1.65) |  |
|  |  |  |  |  | 9901–12800 ml/week | 1.17(0.51–2.72) |  |
|  |  |  |  |  | >12800 ml/week | 0.96(0.42–2.22) |  |
|  |  |  | M:602/615 | M | <8300 ml/week | 1.0 |  |
|  |  |  |  |  | 8301–10400 ml/week | 0.87(0.58–1.30) |  |
|  |  |  |  |  | 10401–12900 ml/week | 1.13(0.76–1.67) |  |
|  |  |  |  |  | 12901–16800 ml/week | 1.41(0.96–2.08) |  |
|  |  |  |  |  | >16800 ml/week | 1.07(0.72–1.59) |  |
| Radosavljevic, 2003 | Serbia | F/M | 130/130 |  | high vs low | 0.96(0.61-1.52) | 6 |
| Baena, 2006 | Spain | M | 74/89 | M | Drink 3 or more glasses of water between meals | 2.74(1.07–7.00) | 6 |
| Michaud, 2007 | Spain | F/M | 397/664 | / | Q1 | 1.0 | 7 |
|  |  |  |  |  | Q2 | 0.67(0.44–1.02) |  |
|  |  |  |  |  | Q3 | 0.72(0.48–1.09) |  |
|  |  |  |  |  | Q4 | 0.68(0.45–1.04) |  |
|  |  |  |  |  | Q5 | 0.62(0.40–0.95) |  |
| Jiang, 2008 | USA | F/M | 1,586/1,586 | Both | Q1 | 1.0 | 6 |
|  |  |  |  |  | Q2 | 0.89(0.70–1.13) |  |
|  |  |  |  |  | Q3 | 0.94(0.75–1.19) |  |
|  |  |  |  |  | Q4 | 0.98(0.77–1.26) |  |
|  |  |  |  | F | Q1 | 1.0 |  |
|  |  |  |  |  | Q2 | 1.09(0.67–1.76) |  |
|  |  |  |  |  | Q3 | 0.93(0.58–1.51) |  |
|  |  |  |  |  | Q4 | 1.19(0.67–2.09) |  |
|  |  |  |  | M | Q1 | 1.0 |  |
|  |  |  |  |  | Q2 | 0.81(0.61–1.07) |  |
|  |  |  |  |  | Q3 | 0.93(0.71–1.23) |  |
|  |  |  |  |  | Q4 | 0.93(0.71–1.24) |  |
| Ahmad and Pervaiz 2010 | Pakistan | F/M | 50/100 |  | ≥10 vs. <10 glasses/day | 0.025 (0.005-0.115) | 6 |
| Hemelt. 2010 | China | F/M | 381/371 | Both | Q1 | 1.0 | 7 |
|  |  |  |  |  | Q2 | 0.50(0.33–0.77 |  |
|  |  |  |  |  | Q3 | 0.71(0.47–1.07 |  |
|  |  |  |  |  | Q4 | 0.65(0.43–0.98 |  |
|  |  |  |  | F | Q1 | 1.0 |  |
|  |  |  |  |  | Q2 | 0.51(0.17–1.49) |  |
|  |  |  |  |  | Q3 | 1.20(0.46–3.13) |  |
|  |  |  |  |  | Q4 | 2.19(0.89–5.38) |  |
|  |  |  |  | M | Q1 | 1.0 |  |
|  |  |  |  |  | Q2 | 0.50(0.31–0.80) |  |
|  |  |  |  |  | Q3 | 0.46(0.28–0.74) |  |
|  |  |  |  |  | Q4 | 0.58(0.37–0.92) |  |
| Zhang, 2010 | China | F/M | 608/607 |  | ≤750 ml/day | 1.0 | 8 |
|  |  |  |  |  | 750-1500 ml/day | 0.91(0.68-1.23) |  |
|  |  |  |  |  | >1500 ml/day | 0.89(0.65-1.22) |  |
| Ahmad et al.2012 | Pakistan | F/M | 55/99 |  | more than 10 glasses per day | 0.116 (0.04-0.33) | 6 |
| Ahmad et al.2012 | Pakistan | F/M | 50/100 |  | more than 10 glasses per day | 0.161 (0.059-0.441) | 6 |
| Wang, 2013 | USA | F/M | 1,007/1,299 |  | <1696 ml/day | 1.0 | 8 |
|  |  |  |  |  | 1696–2215 ml/day | 0.92(0.71–1.18) |  |
|  |  |  |  |  | 2215–2789 ml/day | 0.81(0.62–1.05) |  |
|  |  |  |  |  | >2789 ml/day | 1.41(1.10–1.81) |  |
| Baris, 2016 | USA | F/M | 1079/1287 |  | ≤1.1 L/day | 1.00 | 8 |
|  |  |  |  |  | >1.1–1.5 L/day | 1.07(0.83,1.38) |  |
|  |  |  |  |  | >1.5–2.2 L/day | 1.17(0.91,1.50) |  |
|  |  |  |  |  | >2.2–3.8 L/day | 1.22(0.94,1.59) |  |
|  |  |  |  |  | >3.8 L/day | 1.86(1.23,2.81) |  |
| Both: both gender; Q: quantity level. | | | | | | | |
